# Challenges Faced by Clinicians in the Personalized Treatment Planning: A Literature Review and the First Results of the Russian National Cancer Program

**DOI:** 10.1155/2021/6649771

**Published:** 2021-09-23

**Authors:** P. V. Shegai, P. A. Shatalov, A. A. Zabolotneva, N. A. Falaleeva, S. A. Ivanov, A. D. Kaprin

**Affiliations:** ^1^Federal State Budgetary Institution National Medical Research Radiological Center of the Ministry of Health of the Russian Federation, Kaluga Region, Koroleva Str. 4., Obninsk 249036, Russia; ^2^Pirogov Russian National Research Medical University, Ostrovitianov Str. 1, Moscow 117997, Russia; ^3^A. Tsyb Medical Radiological Research Center–Branch of the Federal State Budgetary Institution National Medical Research Radiological Center of the Ministry of Health of the Russian Federation, Zhukova Str. 10, Kaluga Region, Obninsk 249031, Russia

## Abstract

Advances in cancer molecular profiling have enabled the development of more effective approaches to the diagnosis and personalized treatment of tumors. However, treatment planning has become more labor intensive, requiring hours or even days of clinician effort to optimize an individual patient case in a trial-and-error manner. Lessons learned from the world cancer programs provide insights into ways to develop approaches for the treatment strategy definition which can be introduced into clinical practice. This article highlights the variety of breakthroughs in patients' cancer treatment and some challenges that this field faces now in Russia. In this report, we consider the key characteristics for planning an optimal clinical treatment regimen and which should be included in the algorithm of clinical decision support systems. We discuss the perspectives of implementing artificial intelligence-based systems in cancer treatment planning in Russia.

## 1. Introduction

Over the past 10 years, clinical care and treatment approaches for patients have changed from a “one-size-fits-all” paradigm to precision or personalized medicine based on genomic variants [1]. In 2015, the Precision Medicine Initiative was launched to find out how a person's genetics, environment, and lifestyle can help determine the most effective method to screen, prevent, or treat disease [2]. Personalized medicine is defined as an approach to patients that takes into consideration their genetic characteristics but with attention to their individual preferences and social features, whereas precision medicine refers to a form of medical care that relies substantially on data and analytical information [1]. Precision and personalized medicine have become especially important for the development of specialized treatments for specific subtypes of cancer, based on the measurement and manipulation of key patient genomic and omic data (transcriptomics, metabolomics, proteomics, etc.).

Molecular profiling (MP) of a tumor refers to the evaluation of nucleic acids (DNA and RNA) and/or proteins within an individual patient's cancer using PCR, fluorescence in situ hybridization (FISH), Sanger sequencing, NGS (whole exome, whole genome, or target sequencing), immunohistochemistry (IHC), and others methods [3]. Each of these methods has its advantages and disadvantages, associated primarily with the cost and sensitivity of the study. Predictive associations for conventional antitumor agents are primarily based on the changes in protein expression as determined by IHC. The detection of “druggable” genetic alterations by NGS only leads to the recommendation of recently introduced targeted therapy drugs, which are usually much more expensive. Although IHC and FISH are basic precision medicine tools in everyday practice, NGS approaches have increasingly substituted conventional methods.

The number of druggable tumor-specific molecular aberrations has grown significantly in the last few years, with an important survival benefit obtained from biomarker matching therapies in many cancer types. The US Food and Drug Administration (FDA) has shown support in the precision medicine approach with their approval of targeted drugs since 1998 when trastuzumab was approved for the treatment of HER2-positive breast cancer. In 2019, the FDA approved 11 new drugs to treat different types of cancer: metastatic breast cancer, refractory bladder cancer, mantle cell lymphoma, ROS1-positive non-small-cell lung cancer, and others (https://www.fda.gov/drugs/new-drugs-fda-cders-new-molecular-entities-and-new-therapeutic-biological-products/novel-drug-approvals-2019). The European MedicinesAgency (EMA) approved 6 new medicines for oncologic diseases in 2019 (https://www.ema.europa.eu/en/news-events/therapeutic-areas-latest-updates/cancer). Most of these drugs belong to the biomarker matching treatment. Also, many new drugs are now in the process of development or at the stage of clinical trials.

Many studies indicate that MP-guided therapy can be beneficial in cancer patients [4–6]. However, the results of the SHIVA trial showed no or limited improvement in progression-free survival for patients receiving targeted therapy. The SHIVA trial was compared to clinical outcomes between patients receiving targeted agents, selected based on panel sequencing, and that given conventional chemotherapy, in patients with any type of metastatic solid tumor refractory to standard treatment [7]. Currently, ongoing large randomized trials (e.g., NCI-MATCH and TAPUR) will add arguments to the value of MP-guided cancer treatment.

Several structured reviews of economic evaluations of MP and precision medicine have been published. The majority of studies concluded that the MP-guided therapy was cost effective compared to usual care [8, 9]. Pages et al. showed that molecular diagnosis accounts for only 6% of the cost of molecular-guided therapy per patient. The costs of drugs and hospitalizations are the major cost drivers [10]. In the case of MP-guided therapy, the high cost can be associated with the longer duration of therapy due to increased overall survival and time to treatment failure [11]. Therefore, the precise determination of the target for treatment during molecular profiling is a key step to improve the quality of treatment and reduce the cost of targeted therapy.

The utility of precision medicine to daily clinical practice relies strongly on the availability of efficient tools to translate an individual's data into diagnosis and targeted treatment. The choice of drug should be based on all the relevant medical information from a patient, including genomics and/or proteomics data, integrated with features describing the drug's properties. Personalized approaches to diagnosing clinically significant alterations have the potential to enable rapid administration of the most adequate therapies tuned to individual features of specific tumors.

## 2. Cancer Statistics and Challenges in Cancer Treatment in Russia

The cancer burden continues to grow all over the world, exerting large physical, emotional, and financial pressure on individuals, families, communities, and health systems. The mortality from malignant neoplasms is in the second place in the structure of causes of death of Russians (for both men and women). The share of these causes in total mortality in 2019 was 17.3% in men and 14.8% in women with the largest contribution in Russia of tumors of the trachea, bronchi, and lungs (17%), stomach (9.3%), colon (8.0%), breast (7.4%), and pancreas (6.7%). Over the past 10 years, cancer mortality in Russia has decreased by 2.1% [12]. However, according to the National Center for Health Statistics, the cancer death rate in the United States fell continuously through 2017, resulting in an overall decline of 29%. This progress is driven by long‐term declines in death rates for the 4 main cancers (lung, colorectal, breast, and prostate) [13].

Two major factors contribute significantly to reducing cancer mortality: an increase in early detection of tumors and an increase in treatment effectiveness. In Russia, by January 1, 2020, the percentage of tumors at stages 1 and 2 of the total number of detected neoplasms increased by 8% compared to the last year, while the percentage of tumors at stages 3 and 4 decreased by 7.8%. These data indicate improvement in the early diagnosis capacity of malignant neoplasms in Russia. This was achieved using two approaches: early recognition of symptomatic cancer in patients and identification of asymptomatic disease in an apparently healthy target population (screening).

Introducing new targeted therapies in routine clinical practice has a great impact on reducing cancer mortality. The term “targeted therapy” refers to all those treatments affecting specific molecular targets: genes, proteins, or the tumor environment [14]. Novel types of cancer treatment include targeted therapies via small molecule inhibitors (SMIs) or monoclonal antibodies (MAbs), as well as several types of immunotherapy: checkpoint inhibitory MAbs, chimeric antigen-specific receptor- (CAR-) transfected T-cells (CAR-T-cells), antitumor vaccines, and oncolytic viruses [15]. These novel treatment approaches are based on the MP of tumors and the precision medicine principle [16]. Drug selection is a complex task that requires the interpretation of personal medical and genetic information as well as the chemical characteristics of the drug. A constantly updated list of approved targeted drugs for the treatment of various types of cancer, considering the personal characteristics of the patient, can make the choice of a doctor difficult.

The Federal Project “Fight against cancer” was launched in 2019 by the decree of the President of the Russian Federation. This is of great importance for the Russian healthcare system, as the problems of cancer prevention and treatment were planned as a national priority. Also, its first results allow us to draw the first conclusions. In the framework of this project, state funding for the treatment of malignant neoplasms was more than doubled compared to 2018. This allowed the use of modern targeted and immunological drugs included in international and national clinical guidelines. New antitumor drugs are used not only in national but also in regional cancer centers. The analysis, carried out as part of this project in Russia, showed that the provided annual budget for chemotherapy was used in 2019 by 63.1%. The Russian Federal Project “Fight against cancer” in the first year of implementation showed good results, but we assume the great potential for a decision-making assistance system that will ensure rational and efficient use of finance and increase the mentioned indicator by one and a half time.

## 3. Algorithm of Decision-Making Systems

Treatment planning is an essential step in the cancer therapy workflow. It has become more sophisticated over the past couple of decades with the help of computer science. As a result, treatment planning can take hours and days of the planner's efforts to optimize an individual patient's case by trial and error. Halford and colleagues showed that a structure defined on four variables is at the limit of human processing capacity [17]. However, to determine the appropriate cancer therapy, it is necessary to consider a lot of complex data. Decision support systems are hailed as a convenient solution to the difficult cognitive burden currently placed on clinicians [18]. More recently, artificial intelligence (AI) and machine learning algorithms have been used to automate and improve various aspects of decision support systems [19].

In the computer science field, AI is defined as the study of algorithms and devices that perceive information from the environment and take action to maximize the chance of achieving specific goals. In medicine, the most prevalent application of AI is the prediction of drug therapy for an individual patient, the definition of drug-target or drug-drug interactions, optimization of dosage and drug schedule, and selection of drug combinations [20]. With AI, machine-learning algorithms analyze data and yield knowledge that can support decisions about new unseen data. The algorithms are trained, tuned, and tested on retrospective/prospective data. Then, these models can be used to predict the outcomes (e.g., survival, quality of life, and side effects) of different treatments based on data from a new unseen patient. Ding and colleagues showed that data-driven approaches significantly outperform current rule-based methods using the genomic status of drug targets as therapeutic indicators [21].

The drug efficiency depends significantly on several characteristics: tumor characteristics (type, localization, tumor size, and stage), personal factors (age, race, sex, family history, other diseases, and environmental factors), prior therapy, and molecular signature of the tumor. A decision support system that can amass all this information holds promise to provide precise classification and guide treatment choices. Better prediction of the effectiveness of targeted therapies can increase the use of new targeted drugs for cancer patients. Accurate prediction of the effectiveness of nonspecific treatments can reduce the prescription of those drugs to patients for whom they will not be effective, leading to improvements in cost and quality of life for patients who would otherwise only suffer through a toxic, ineffective first-line regimen.

Further in this article, we provide the fundamental characteristics, which should be included in the clinical decision-making algorithm. Each feature is provided with an example from the list of new drugs approved by the FDA or EMA.

### 3.1. Clinicopathological Parameters

#### 3.1.1. Tumor Characteristics (Type, Size, Localization, and Stage)

There are over 100 types of human cancers, locating in different organs and tissues and originating from different cell types. Most targeted drugs are recommended for treating patients with certain types and stages of cancer. The action of some anticancer drugs was shown to be organ specific, which is associated with tumor metabolism [22].

Treatment for cancer at stage 1 or 2 is often different from treatment for the same type of cancer at a late stage. Early diagnosis and complete cancer registry are fundamental issues in the better prognosis and survival of cancer patients. Early detection of cancer is also associated with more effective and less complex therapy as well as lower costs of treatment [23, 24].

Several studies have shown a correlation between tumor size or grade and treatment choice or response to treatment. However, the prognostic value of these characteristics for different types of cancer may vary. For most solid tumors, greater tumor size, metastases, higher clinical disease stage, and the histological grade of differentiation were found to be significant unfavorable prognostic indicators of overall survival [25–27]. Exceptions are observed among some types of cancer; for example, the small tumor size in patients with locally advanced laryngeal cancer (T4) or regionally (N+) could be regarded as an unfavorable prognostic factor of overall survival [28]. In addition, the size of the tumor (or the number of cells, in the case of liquid tumors) at diagnosis can affect the development of drug resistance. According to the Goldie–Coldman hypothesis, the probability that cancer contains drug-resistant clones depends on the tumor size and the mutation rate [29].

Therapy case: Polivy (polatuzumab vedotin-piiq; Genentech Inc., USA) is used to treat adults with one type of tumor: diffuse large B-cell lymphoma. Turalio (pexidartinib; Daiichi Sankyo Inc., Japan) is a drug used to treat adults with a tumor in the protective layer surrounding the tendons (tenosynovial giant cell tumor).

#### 3.1.2. Personal Factors of the Patient

The actual administration of a first-line treatment often considers the patient's age (current and at diagnosis), weight, race, gender, family history, other diseases, history of smoking, environmental factors, etc. These characteristics can affect both the efficiency of cancer therapy and the risk of side effects. Many studies have demonstrated the impact of personal characteristics on the absorption, distribution, metabolism, and elimination of drugs in the body [30]. Elderly and pediatric patients are particularly vulnerable to adverse reactions because drugs are less likely to be studied extensively in these ages and drug characteristics are more variable and less predictable in both cohorts [31, 32]. The presence of concomitant diseases in the patients can also increase susceptibility to adverse drug effects [33]. This suggests that several personal factors should be considered before selecting a cancer treatment.

Therapy case: Rozlytrek (entrectinib; Genentech Inc., USA) was recommended for adult and adolescent patients (12 to 17 years old) with non-small-cell lung cancer (NSCLC). It is recommended to assess left ventricular ejection fraction before initiation of this drug in patients with congestive heart failure.

#### 3.1.3. The Presence of Prior Therapy

First-line therapy is the initial treatment that was accepted as the best treatment for certain types and stages of cancer. This therapy may include over one method: chemotherapy, surgical treatment, or radiotherapy for different tumor types and stages. Many targeted drugs and immunotherapy are used as second-line therapy or further lines of therapy (third-line, fourth-line, seventh-line, etc.).

Therapy case: Brukinsa (zanubrutinib; BeiGene, China) capsules were recommended for the treatment of patients with mantle cell lymphoma who have received at least one prior therapy. Nubeqa (darolutamide; Bayer, Germany) is approved for the treatment of prostate cancer that has not spread to other parts of the body (nonmetastatic) and no longer responds to medical or surgical treatment that lowers testosterone (castration resistant).

#### 3.1.4. MP Results

Many experiments have collected genomic, transcriptomic, and proteomic data on numerous cancer cell lines and patient-derived xenografts, together with drug sensitivity data. Therefore, anticancer agents are increasingly being combined with genetic alterations and biomarkers to determine which patients are most likely to benefit from the therapy.

Many studies indicate that thousands of genes are associated with the subtype and prognosis of cancer, and specific allele combinations may usefully guide the treatment selection [34]. For example, the 2nd generation of EGFR-tyrosine kinase inhibitors has demonstrated activity against tumors with T790M mutation in EGFR (Epidermal Growth Factor Receptor), but they also irreversibly inhibit wild-type EGFR, causing severe toxic side effects. Therefore, the 3rd generation of EGFR-tyrosine kinase inhibitors (rociletinib (CO-1686; Clovis Oncology, USA), osimertinib (AZD9291/Tagrisso®; AstraZeneca; formerly mereletinib), olmutinib (HM61713; Hanmi Pharmaceutical, South Korea), tesevatinib (XL647/KD019; Kadmon Corporation, USA), naquotinib (ASP8273; Astellas Pharma Inc., Japan), etc., is in active clinical development to target only EGFR-T790M tumors [35].

Genetic mutations affect not only treatment selection but also the response of specific cancer to therapy. The specific mutations in the specific genes are correlated with effectiveness, clinical responsiveness, and resistance to the targeted therapy. For example, 85 to 90% of patients with EGFR therapy resistance had mutations in KRAS codons 12 and 13 (exon 2) [36].

There are several models to predict the response of cancer cell lines to drug treatment, quantified through IC_50_ values based on both the genomic features (mutation profiles, microsatellite instability, and copy number alterations) and the chemical properties of the drugs [37, 38].

Therapy case: Piqray (alpelisib; Novartis Pharmaceuticals Corporation, Switzerland) was recommended to treat postmenopausal women and men with hormone receptor- (HR-) positive human epidermal growth factor receptor 2- (HER2-) negative, PIK3CA-mutated, advanced, or metastatic breast cancer following progression on or after an endocrine-based regimen.

The decision-making system in oncology should not only determine the most suitable drug based on patient data but also consider the benefits of a combination of drugs, as well as determine the dose and regimen of the drug.

#### 3.1.5. Combination Therapy

The combination of two or more therapeutic methods to specifically or nonspecifically reach high rates of tumor cell eradication is a cornerstone of cancer treatment. Some well-combined treatments are known. For example, oncolytic virus therapy can be combined with immune checkpoint blockade (ICB), SMIs, radiotherapy, and adoptive T-cell immunotherapy [39].

Regarding chemotherapy drugs, the administration of a single drug with specific molecular targets may prove insufficient for the treatment of the most aggressive tumors, while knowing all driver mutations, one can prescribe a more effective combined therapy [40]. The drawback of monotherapy is the high probability that tumor cells will develop resistance to the drug, with subsequent proliferation and repopulation of the tumor [41].

However, simultaneous administration can cause no interaction between drugs and, thus, no net beneficial effect or adverse interactions, leading to decreased efficiency and possible toxicity.

Synergistic combinations are drugs that amplify each other's activity, leading to elevated effects at low concentrations and, thus, reduced toxicity. It has been demonstrated that synergism predictions are significantly more dependent on drug features, chemical descriptors, similarity metrics, and interaction networks than on patient features and genetic profiles [42]. There are several computational models for the prediction of synergism and antagonism between oncological drugs [43–45].

Predicting drug-drug interactions is also important to the drug treatment selection and administration process because it can help to minimize adverse reactions and healthcare costs, as well as maximize dosage efficiency. Many of these interactions are screened as part of the FDA approval process, but some of them go unnoticed until after clinical trials due to the enormous number of possible combinations. The computational prediction may assist in identifying potential drug-drug interactions [45]. Several AI-based models were developed for the a priori detection of drug-drug interactions from biological, chemical, and pharmacokinetic data with high accuracy in the academic setting, but none have reached the clinical implementation stage [46, 47].

Implementing known synergistic drug combinations or a computational model for the prediction of synergism or drug-drug interactions in the decision-making system will significantly improve the quality of cancer therapy.

Therapy case: Xpovio (selinexor; Karyopharm Therapeutics Inc., USA) was approved as a treatment for patients with multiple myeloma in combination with dexamethasone. This combination was specified by the manufacturer. AI can be used to predict other drug interactions.

#### 3.1.6. Dosage

An ideal dosage regimen keeps the concentration of drug in the body at a constant equilibrium above the minimum effective concentration but below the minimum toxic concentration. The dose-response curve is the most important difference between biological cancer therapies and cytostatic drugs, while this curve is linear for cytostatic drugs, and it is bell-shaped for biological and physiological therapies [15]. One possible reason for this shape of the dose-response curve in the case of biological and physiological approaches is molecular complementarity [39]. Several computer modeling-based approaches are used to optimize schedule cancer chemotherapy to ensure effective and safe treatment [48].

Therefore, the calculation of the appropriate dose of the selected drug should be included in the algorithm for making a clinical decision for an individual patient.

## 4. Conclusions

Precision oncology can and should be a practice of effectively utilizing all available approaches, including molecularly targeted, immunotherapy, and cytotoxic chemotherapy in a patient-specific manner. To determine the cancer therapy most suitable for the patient, it is necessary to consider a lot of complex genetic, medical, and chemical data; therefore, the use of modeling methods relied upon in computational tools, including machine learning and AI technologies, is necessary for a decision-making process. Only through the application of a computer-based decision support system can we truly leverage the vast amount of patient data that needs to be integrated to fully understand and fight cancers. The purpose of computational tools and algorithms in drug decision is to reduce the data to a size the doctor can interpret, giving clinicians access to information they could not previously consider. These clinical support systems can not only improve healthcare system services, decision timing, medical error rates, and health-related quality of life for patients but also reduce healthcare costs.

## Data Availability

No data were used to support this study.

## Abbreviations

AI: Artificial intelligence
EGFR: Epidermal growth factor receptor
EMA: European Medicines Agency
FDA: Food and Drug Administration
FISH: Fluorescence *in situ* hybridization
ICB: Immune checkpoint blockade
IHC: Immunohistochemistry
MAbs: Monoclonal antibodies
MP: Molecular profiling
NGS: Next-generation sequencing
NSCLC: Non-small-cell lung cancer
SMIs: Small-molecule inhibitors.

## References

[1] Ginsburg G. S., Phillips K. A. (2018). Precision medicine: from science to value. *Health Affairs*.

[2] Collins F. S., Varmus H. (2015). A new initiative on precision medicine. *New England Journal of Medicine*.

[3] El-Deiry W. S., Goldberg R. M., Lenz H. J., Shields A. F., Gibney G. T., Tan A. R. (2019). The current state of molecular testing in the treatment of patients with solid tumors. *CA Cancer Journal Clinical 2019*.

[4] Stockley T. L., Oza A. M., Berman H. K. (2016). Molecular profiling of advanced solid tumors and patient outcomes with genotype-matched clinical trials: the Princess Margaret IMPACT/COMPACT trial. *Genome Medicine*.

[5] Epelbaum R., Shacham-Shmueli E., Klein B. (2015). Molecular profiling-selected therapy for treatment of advanced pancreaticobiliary cancer: a retrospective multicenter study. *BioMed Research International*.

[6] Oliver K. E., Xiao N., Spetzler D., Phippen N. T., Oleszewski R. T., McGuire W. P. (2014). The impact of tumor molecular profile-directed treatment on survival in recurrent ovarian cancer. *Journal of Clinical Oncology*.

[7] Le Tourneau C., Delord J.-P., Gonçalves A. (2015). Molecularly targeted therapy based on tumour molecular profiling versus conventional therapy for advanced cancer (SHIVA): a multicentre, open-label, proof-of-concept, randomised, controlled phase 2 trial. *The Lancet Oncology*.

[8] Spizzo G., Siebert U., Gastl G. (2019). Cost-comparison analysis of a multiplatform tumour profiling service to guide advanced cancer treatment. *Cost Effectiveness and Resource Allocation*.

[9] Henderson R., French D., Sullivan R., Maughan T., Clarke M., Lawler M. (2019). Molecular biomarkers and precision medicine in colorectal cancer: a systematic review of health economic analyses. *Oncotarget*.

[10] Pagès A., Foulon S., Zou Z. (2017). The cost of molecular-guided therapy in oncology: a prospective cost study alongside the MOSCATO trial. *Genetics in Medicine*.

[11] Chawla A., Janku F., Wheler J. J. (2018). Estimated cost of anticancer therapy directed by comprehensive genomic profiling in a single-center study. *JCO Precision Oncology*.

[12] Kaprin A. D., Starinsky V. V., Petrova G. V. (2019). *Malignant Neoplasms in Russia in 2018 (Incidence and Mortality)*.

[13] Siegel R. L., Miller K. D., Jemal A. (2020). Cancer statistics, 2020. *CA: A Cancer Journal for Clinicians*.

[14] Tsimberidou A.-M. (2015). Targeted therapy in cancer. *Cancer Chemotherapy and Pharmacology*.

[15] Schirrmacher V. (2017). *Quo Vadis Cancer Therapy? Fascinating Discoveries of the Last 60 Years*.

[16] Krzyszczyk P., Acevedo A., Davidoff E. J. (2018). The growing role of precision and personalized medicine for cancer treatment. *Technology*.

[17] Halford G. S., Baker R., McCredden J. E., Bain J. D. (2005). How many variables can humans process?. *Psychological Science*.

[18] Walsh S., de Jong E. E. C., van Timmeren J. E. (2019). Decision support systems in oncology. *JCO Clinical Cancer Informatics*.

[19] Davenport T., Kalakota R. (2019). The potential for artificial intelligence in healthcare. *Future Healthcare Journal*.

[20] Romm E. L., Tsigelny I. F. (2020). Artificial intelligence in drug treatment. *Annual Review of Pharmacology and Toxicology*.

[21] Ding M. Q., Chen L., Cooper G. F., Young J. D., Lu X. (2018). Precision oncology beyond targeted therapy: combining omics data with machine learning matches the majority of cancer cells to effective therapeutics. *Molecular Cancer Research*.

[22] Elia I., Schmieder R., Christen S., Fendt S. M. (2016). Organ-specific cancer metabolism and its potential for therapy. *Handbook of Experimental Pharmacology*.

[23] Campbell J. D., Ramsey S. D. (2009). The costs of treating breast cancer in the US. *PharmacoEconomics*.

[24] Blumen H., Fitch K., Polkus V. (2016). Comparison of treatment costs for breast cancer, by tumor stage and type of service. *American Health & Drug Benefits*.

[25] Jang J. Y., Choi N., Ko Y.-H. (2018). Treatment outcomes in metastatic and localized high-grade salivary gland cancer: high chance of cure with surgery and post-operative radiation in T1-2 N0 high-grade salivary gland cancer. *BMC Cancer*.

[26] McHugh C. H., Roberts D. B., El-Naggar A. K. (2012). Prognostic factors in mucoepidermoid carcinoma of the salivary glands. *Cancer*.

[27] Yasukawa M., Sawabata N., Kawaguchi T. (2018). Histological grade: analysis of prognosis of non-small cell lung cancer after complete resection. *In Vivo*.

[28] Markou K., Goudakos J., Triaridis S., Konstantinidis J., Vital V., Nikolaou A. (2011). The role of tumor size and patient’s age as prognostic factors in laryngeal cancer. *Hippokratia*.

[29] Goldi J. H., Coldman A. J. (1979). A mathematic model for relating the drug sensitivity of tumors to their spontaneous mutation rate. *Cancer Treatment Reports*.

[30] Hartmanshenn C., Scherholz M., Androulakis I. P. (2016). Physiologically-based pharmacokinetic models: approaches for enabling personalized medicine. *Journal of Pharmacokinetics and Pharmacodynamics*.

[31] Rieder M. (2019). Adverse drug reactions in children: pediatric pharmacy and drug safety. *Journal of Pediatric Pharmacology and Therapeutics*.

[32] Sneha S. G., Simhadri K., Subeesh V. K., Sneha S. V. (2019). Predictors of adverse drug reactions in geriatric patients: an exploratory study among cancer patients. *South Asian Journal of Cancer*.

[33] Alomar M. J. (2014). Factors affecting the development of adverse drug reactions (Review article). *Saudi Pharmaceutical Journal*.

[34] Neve R. M., Chin K., Fridlyand J. (2006). A collection of breast cancer cell lines for the study of functionally distinct cancer subtypes. *Cancer Cell*.

[35] Seebacher N. A., Stacy A. E., Porter G. M., Merlot A. M. (2019). Clinical development of targeted and immune based anti-cancer therapies. *Journal of Experimental & Clinical Cancer Research*.

[36] Misale S., Di Nicolantonio F., Sartore-Bianchi A., Siena S., Bardelli A. (2014). Resistance to anti-EGFR therapy in colorectal cancer: from heterogeneity to convergent evolution. *Cancer Discovery*.

[37] Menden M. P., Iorio F., Garnett M. (2013). Machine learning prediction of cancer cell sensitivity to drugs based on genomic and chemical properties. *PLoS One*.

[38] Chang Y., Park H., Yang H.-J. (2018). Cancer drug response profile scan (CDRscan): a deep learning model that predicts drug effectiveness from cancer genomic signature. *Scientific Reports*.

[39] Schirrmacher V. (2019). From chemotherapy to biological therapy: a review of novel concepts to reduce the side effects of systemic cancer treatment (Review). *International Journal of Oncology*.

[40] Zsákai L., Sipos A., Dobos J. (2019). Targeted drug combination therapy design based on driver genes. *Oncotarget*.

[41] Vasan N., Baselga J., Hyman D. M. (2019). A view on drug resistance in cancer. *Nature*.

[42] Tsigelny I. F. (2019). Artificial intelligence in drug combination therapy. *Briefings in Bioinformatics*.

[43] Li X., Xu Y., Cui H. (2017). Prediction of synergistic anti-cancer drug combinations based on drug target network and drug induced gene expression profiles. *Artificial Intelligence in Medicine*.

[44] Xia F., Shukla M., Brettin T. (2018). Predicting tumor cell line response to drug pairs with deep learning. *BMC Bioinformatics*.

[45] Celebi R., Bear Don’t Walk O., Movva R., Alpsoy S., Dumontier M. (2019). In-silico prediction of synergistic anti-cancer drug combinations using multi-omics data. *Scientific Reports*.

[46] Cheng F., Zhao Z. (2014). Machine learning-based prediction of drug-drug interactions by integrating drug phenotypic, therapeutic, chemical, and genomic properties. *Journal of the American Medical Informatics Association*.

[47] Kastrin A., Ferk P., Leskošek B. (2018). Predicting potential drug-drug interactions on topological and semantic similarity features using statistical learning. *PLoS One*.

[48] Padmanabhan R., Meskin N., Haddad W. M. (2017). Reinforcement learning-based control of drug dosing for cancer chemotherapy treatment. *Mathematical Biosciences*.

